# Prevention of COVID-19 in Internally Displaced Persons Camps in War-Torn North Kivu, Democratic Republic of the Congo: A Mixed-Methods Study

**DOI:** 10.9745/GHSP-D-20-00272

**Published:** 2020-12-23

**Authors:** Kasereka M. Claude, Muyisa Sahika Serge, Kahindo Kahatane Alexis, Michael T. Hawkes

**Affiliations:** a Department of Medicine, Université Catholique du Graben, Butembo, Democratic Republic of the Congo.; b Department of Ophthalmology, Université Catholique du Graben, Butembo, Democratic Republic of the Congo.; c Department of Pediatrics, University of Alberta, Edmonton, Canada.; d Department of Medical Microbiology and Immunology, University of Alberta, Edmonton, Canada.; e School of Public Health, University of Alberta, Edmonton, Canada.; f Stollery Science Lab, University of Alberta, Edmonton, Canada.; g Women and Children's Research Institute, University of Alberta, Edmonton, Canada.

## Abstract

**Background:**

The coronavirus disease (COVID-19) pandemic poses a grave threat to refugees and internally displaced persons (IDPs). We examined knowledge, attitudes, and practices with respect to COVID-19 prevention among IDPs in war-torn Eastern Democratic Republic of the Congo (DRC).

**Methods:**

Mixed-methods study with qualitative (focus group discussions, [FGDs]) and quantitative (52-item survey questionnaire) data collection and synthesis.

**Results:**

FGDs (N=23) and survey questionnaires (N=164 IDPs; N=143 comparison group) were conducted in May 2020. FGD participants provided narratives of violence that they had fled. IDPs were statistically more likely to have larger household size, experience more extreme poverty, have lower educational attainment, and have less access to information through media and internet versus the comparison group (*P*<.05 for the comparison group). IDPs had a high level of awareness (99%) and fear (98%) of COVID-19, but lower specific knowledge (15% sufficient knowledge versus 30% among the comparison group, *P*<.0001), a difference which remained significant in a multivariable model adjusting for confounding. IDPs faced major barriers to implementing COVID-19 prevention measures. Physical distancing was impossible for IDPs in crowded shelters, and 70% reported coming in close contact with someone other than a family member within the past 24 hours (versus 56% of the comparison group, *P*=.014). Frequent movements in and out of the camp for subsistence left IDPs vulnerable to the introduction of COVID-19: 61% left the camp on a daily basis and 65% had received a visitor in the past month. Despite acceptance of hand hygiene for prevention, 92% lacked soap (versus 65% of the comparison group, *P*<.0001). IDPs’ desire for peace and to return to their native homes, where COVID-19 precautions could be feasibly implemented, overshadowed their perceived benefits of measures such as a COVID-19 vaccine.

**Conclusions:**

These findings provide empiric evidence supporting the vulnerability of IDPs to COVID-19 and call for action to protect neglected displaced populations.

## Abstract (precis)

Internally displaced persons fleeing violent conflict represent a neglected population with heightened vulnerability to pandemic COVID-19. We provide a rare snapshot of the overwhelming challenges faced by internally displaced persons in Eastern Democratic Republic of the Congo as they brace for COVID-19.

## Teaser

**Key Findings:**

- Congolese internally displaced persons (IDPs) had high awareness and fear of COVID-19, but low specific knowledge.
- IDPs face major barriers to implementing COVID-19 prevention measures: crowded shelters, frequent movements in and out of the camp for work, and lack of soap for hand hygiene.
- IDPs’ desire for peace and to return to their native homes, where COVID-19 precautions could be feasibly implemented, overshadowed their enthusiasm for other control measures such as a vaccine.

**Key Implications:**

- Donors and policy makers should consider providing consumables, such as soap for hand hygiene and face masks, to implement COVID-19 precautions.
- The national government or international aid agencies should consider providing individual family dwellings (e.g., tarpaulin tents) to allow IDPs to practice physical distancing.
- National and international governments should take serious measures to restore peace to the area by controlling armed conflict. A safe return to their homes would allow IDPs to practice COVID-19 prevention without external aid.

Résumé en français à la fin de l'article.

## INTRODUCTION

As of August 25, 2020, there have been more than 24 million cases of coronavirus disease (COVID-19) confirmed worldwide and 800,000 deaths, with the United States and Europe experiencing the highest burden.^1^ African countries have reported 298,000 cases and 8,000 deaths (case fatality ratio 2.4%).^2^ Many low-resource settings lack comprehensive surveillance and laboratory testing to monitor the spread of COVID-19.^3^ The presence of displaced populations (refugees and internally displaced persons [IDPs]) adds further complexity to the COVID-19 pandemic and control measures in low-and middle-income countries (LMICs) in conflict zones.

In the Democratic Republic of the Congo (DRC), the first case of COVID-19 was detected on March 10, 2020, in a traveler returning from France.^4^ Since then, more than 9,800 cases and 251 deaths have been confirmed across the DRC. Most cases have been detected in the capital city, Kinshasa. In the province of North Kivu, there have been 203 cases as of August 25, 2020. The primary mode of transmission is community based.^5^ In response to the pandemic, the government declared a state of public health emergency on March 24, 2020, with broad closure of businesses, gatherings, and travel.^4^ Since this initial lockdown, the government authorized gradual reopening of businesses and public transportation (July 22); schools and universities (August 3); and churches, interprovincial travel, and international airports (August 15).^4^

Refugees and migrants are among the world’s most vulnerable people.^6^ Worldwide, there are approximately 26 million refugees and 46 million IDPs, displaced due to insecurity and natural disasters.^7^ The DRC has the second highest number of IDPs of any country in the world (after Syria), estimated at more than 5.5 million.^8^ Displaced populations, housed in temporary shelters or camps, generally have limited access to quality shelter, sanitation, clean water, stable food supply, and health care. Under these conditions, COVID-19 prevention efforts may be challenging.^9^ ^–^ ^11^

Impacts of the COVID-19 pandemic on displaced populations are predicted to be disastrous. Already, resettlement procedures have been suspended by the United Nations, alongside widespread travel bans. The first case of COVID-19 in the island of Lesvos in March 2020 raised the alarm for the 20,000 residents of the Moria refugee camp, where distancing is a physical impossibility.^9^ In the world's largest refugee camp in Bangladesh, which shelters more than 855,000 Rohingya refugees, preparations for COVID-19 have begun, such as portable handwashing facilities at every community center.^12^ In Nigeria, efforts to mitigate the impacts of COVID-19 have included sensitization campaigns on handwashing and distribution of soap to more than 100,000 IDPs.^13^ However, as noted by previous authors, recommendations for hand hygiene and physical distancing may be extremely difficult to implement in a refugee or IDP camp. How do you self-isolate in a refugee camp?^10^ Several commentators have forewarned of an impending crisis if COVID-19 strikes in refugee or IDP camps.^6^ ^,^ ^10^ ^,^ ^12^ However, a paucity of empirical data from these areas is available.

Our overarching goal was to contribute to the improvement of prevention strategies against COVID-9 in IDP camps in the DRC. We aimed to describe the knowledge, attitudes, and practices (KAPs) of IDPs in Eastern DRC with respect to the prevention of COVID-19. Our primary endpoint was COVID-19 specific knowledge, which we compared between IDPs and individuals from neighboring villages. Other specific objectives included: (1) to describe attitudes of IDPs with respect to COVID-19 and its prevention; (2) to describe the practices used by IDPs for preventing COVID-19; and (3) to describe barriers faced by IDPs in implementing recommended COVID-19 prevention measures.

Using qualitative and quantitative methods, we aimed to provide rich data on a highly vulnerable and neglected group facing the COVID-19 pandemic in an environment of extreme scarcity and insecurity.

## METHODS

### Study Design

We conducted a mixed-methods study with qualitative focus group discussion (FGDs) and quantitative (52-item survey questionnaire) data collection. Mixed-methods research seeks to triangulate data from qualitative and quantitative methods.^14^ Convergence of findings from multiple methods may enhance the validity of results (multiple operationalism).^15^ We and others have previously used this methodology to integrate community attitudes, behaviors, and responses into epidemiological research.^16^ ^,^ ^17^ With respect to the survey questionnaire, the study followed a descriptive cross-sectional design.

### Study Setting

The province of North Kivu has a population of 6.7 million inhabitants and an estimated 1.7 million IDPs.^18^ The Eastern provinces of the DRC have been the arena of a complex humanitarian emergency for several decades. Mortality rates are 70% above pre-war levels, due largely to preventable and treatable infectious diseases rather than the direct effects of conflict.^19^ Large-scale population displacement has resulted in numerous IDP camps throughout the area.^20^ The chronic threats to security have long been neglected by the national government and the international community.^17^

We selected 3 IDP camps (Mwangaza, Masosi, and Luvangira) located 2 to 5 km from the rural commune of Oïcha, North Kivu. These temporary settlements consisted of groups of IDPs sheltered in school buildings or mud/thatch dwellings on public grounds.^11^ Camp census data indicated the following populations: Mwangaza (1064 individuals, 200 households); Masosi (869 individuals, 176 households); and Luvangira (250 individuals, 75 households). Aid for the camp is coordinated by the nongovernmental organization Charité Aide et Développement, Axe Oïcha, with intermittent assistance from OXFAM,^21^ World Food Programme,^22^ and International Committee of the Red Cross.^23^

### FGDs

Participants of FGDs were purposively selected from the 3 IDP camps. Participants included adult women (3 FGDs) and men (2 FGDs) who were heads of households, and youth (1 FGD). FGDs were conducted in Congolese Swahili. Discussions were recorded, translated, and transcribed into English for subsequent analysis. FGDs lasted 30–45 minutes and included 3 or 4 participants in each group.^24^ The FGD topic guide was adaptive, allowing us to confirm findings and explore emerging themes from each FGD session. Questions were open-ended and elastic, allowing participants to shape the discussion. FGDs were continued until saturation.^25^ Thematic analysis was used to identify, analyze, and report themes in the FGDs.^26^ Two investigators (KMC and MH) read the transcripts several times, noted preliminary ideas, produced initial codes, then generated and refined themes. Representative quotations as well as statements of particular interest were extracted to support the themes.

### Survey Questionnaire

We developed a 52-item questionnaire based on past COVID-19 questionnaires used in Guyana^27^ and Uganda.^28^ The choice of questionnaire items was guided by a need for contextually appropriate questions for low-income settings. We also drew on past experience from past surveys conducted in IDP camps in the area^29^ ^–^ ^32^ and from the recent Ebola virus disease epidemic^16^ ^,^ ^17^ ^,^ ^33^ to design questions that would be relevant and understood by the participants. A local Congolese physician (KMC) with tacit knowledge of the circumstances, culture, and language of the IDPs chose the appropriate wording of the questions and adapted the content of the questionnaire to the conditions in the IDP camp. The survey was administered to IDPs in the 3 camps as follows.

### Sampling

#### Statistical Unit and Estimation of Sample Size

The unit of analysis was the household, defined as a family unit, often consisting of male and female parents and their children.

For our primary analysis, we focused on differences in sufficient knowledge (binary variable) between IDPs and the comparison group. A standard sample size calculation indicated that 138 households would be needed to detect a difference of 15%, with 95% confidence and 80% power, assuming that the proportion of IDPs with sufficient knowledge was 20% or less, based on our previous study of knowledge of Ebola virus disease among IDPs.^33^

#### Sampling Technique

Geospatial sampling^34^ was used, as in previous studies of mobile populations. IDP camps were divided geographically into thirds and 1 area was chosen at random. All households living within the selected area were included, and the standardized questionnaire was administered to 1 adult member from each household. Our sampling technique was inspired by the cluster sampling method developed by the WHO for monitoring vaccine coverage.^35^ ^,^ ^36^ In this approach, a population is divided into a specified number of geographic “clusters” (in our case, camps) of a known or estimable population size. Within each cluster, the desired number of households are selected (in our case, approximately one-third were needed to reach the required sample size).^36^ Several strategies are possible for household selection (e.g., enumeration of all households and simple random sampling from this list, or a “random walk” sampling contiguous households).^36^ However, random selection in more densely populated areas (e.g., urban settings or, in our case, an IDP camp) can be more challenging, given the more complex household types (e.g., apartment buildings or, in our case, IDPs sheltering in school classrooms). In such settings, a common approach is to divide the geographic area of interest into zones, randomly select a zone, and randomly select a starting point within that zone. To reach our desired sample size, we needed to sample approximately one-third of the camp households. Therefore, we chose to divide the camp into thirds, choose 1 cluster at random, and sample all households within that cluster. For our comparison group, we surveyed the surrounding villages (nondisplaced population) using a nonprobability, purposive, maximum variation sampling technique,^37^ choosing participants from all demographic categories (men and women, full age spectrum, employment category, education attainment, and marital status). Participants were aged 18 years or older.

### List of Variables

The questionnaire consisted of several domains related to participant demographics, knowledge of COVID-19, attitudes, and behaviors for preventing COVID-19.

#### Demographics

Individual respondent characteristics were collected: age, sex, educational attainment, and marital status. In addition, we collected data on household characteristics (number of family members, members aged 60 years and older) and wealth indicators (ownership of radio, cellular telephone, and bicycle).

#### Knowledge of COVID-19 Symptoms

Participants were asked to choose from a list of possible sources they drew upon for information on COVID-19 (multiple selections possible). Using a list of symptoms, including 2 detractor (false) symptoms (constipation and bleeding), participants were asked to agree whether COVID-19 was associated with each symptom (“yes” or “no”). Recognition of asymptomatic transmission^38^ ^,^ ^39^ was assessed with the question: “A person who is not sick and who has no symptoms can still spread the virus” (responses: “true,” false,” or “I don’t know”). Agreement with common misconceptions (transmission by mosquitos, prevention with spicy food) was assessed (responses: “yes,” “no,” or “I don't know”).

Participants were considered to have sufficient knowledge of COVID-19 if they identified at least 1 of the cardinal signs and symptoms of COVID-19 (fever, cough, or difficulty breathing),^40^ recognized the potential for asymptomatic transmission, and rejected misconceptions (bleeding as symptom, transmission by mosquitos).

#### Attitudes

We probed a range of attitudes related to COVID-19 by assessing agreement with statements on a 5-point Likert scale (“strongly agree,” “somewhat agree,” “neutral,” “somewhat disagree,” and “disagree,” with a possible “I don't know” response). Affective response was measured using 2 questions about perceived severity and fear of COVID-19. We assessed attitudes toward recommended control measures, including physical distancing and staying home without working. Mistrust and rumors contributed to community resistance to control measures during the recent Ebolavirus epidemic in the DRC.^17^ ^,^ ^41^ Therefore, we included measures of institutional trust (2 items) and endorsement of conspiracies related to the SARS-CoV-2 virus (2 items).

#### Practices

Participants indicated whether they had taken any action to prevent COVID-19 (“yes” or “no”). Among those who answered affirmatively, action(s) they had taken were chosen from a list of possible prevention methods (multiple responses permitted). With respect to physical distancing, we inquired whether the participant had come in close contact with someone outside the family (responses: “yes” or “no”) and with how many people they had shaken hands in the past 24 hours (responses: “none,” “1 to 5,” “more than 5”). Participants selected 1 or more barriers to COVID-19 prevention from a list of possible barriers (multiple responses permitted, with an option to respond “I can fully protect myself against COVID-19”).

### Data Collection Technique

The standardized questionnaire was administered as a verbal structured interview, with a study team member asking questions in the local language and recording the participant's answers using a field-adapted electronic data collection tool, KoboToolbox.^42^ Study team members were local Congolese health workers with tacit understanding of the language and culture, biomedical understanding of COVID-19, and past experience administering surveys by verbal interview.

### Data Processing and Analysis

For descriptive statistics, we used median and interquartile range for continuous variables, and number (percentage) for proportions. Comparative statistics were computed using non-parametric methods: Mann-Whitney U-test for continuous variables and Chi squared or Fisher’s exact test for dichotomous variables, as appropriate. With respect to our primary analysis, we expressed the association between IDP status and knowledge as odds ratio (OR), the cross-product ratio of the entries in the 2-by-2 contingency table of 2 binary variables.^43^ Multivariable logistic regression was used to verify the association between IDP status and knowledge, with adjustment for confounding variables. Statistical analysis was performed in the R statistical environment.^44^

### Ethics Considerations

Participants provided verbal consent to participate in the FGD and the questionnaire. Ethics approval was obtained from the Comité d’Éthique du Nord Kivu (Université Catholique du Graben, ref 003/TEN/CENK/2020). Operational approval was granted by the municipal authority (bourgmestre) and the local refugee coordinator. Participant confidentiality was respected during implementation and analysis of survey results. Data were collected anonymously, without identifiers, and all results were presented in aggregate so that no individual participant can be identified. All names and locations were removed from FGD quotations to avoid possible identification of the speaker.

## RESULTS

### FGD Themes

We began with a qualitative exploration of COVID-19 prevention in the IDP camp. We conducted 6 FGDs, involving 23 participants (total). The composition of focus groups is shown in Table 1. FGDs generated rich qualitative data, from which we derived the following themes: (1) displacement narratives; (2) population movements in and out of the camp and risk of introducing COVID-19; (3) high level of awareness and fear of COVID-19; (4) challenges associated with hand hygiene in the camp; (5) impossibility of physical distancing in the IDP camp; and (6) restoring peace and security takes priority over vaccine.

**TABLE 1.** Composition of Focus Groups From 3 Internally Displaced Persons Camps in North Kivu, Democratic Republic of the Congo

**Focus Group**	**IDP Participants**	**Location**	**Participant Unique Identifier**
1	4 men	Mwangaza	M1, M2, M3, M4
2	4 women	Masosi	F1, F2, F3, F4
3	4 men	Mwangaza	M5, M6, M7, M8
4	3 youth (male)	Masosi	Y1, Y2, Y3
5	4 men	Mwangaza	F5, F6, F7, F8
6	4 women	Luvangira	F9, F10, F11, F12

Abbreviation: IDP, internally displaced person.

We elaborate on each theme and provide representative quotations.

#### Displacement Narratives

Unspeakable terror and killings drove FGD participants from their native homes.

*I've been in this camp for 6 years, since the beginning of the massacres in the region. —*FGD1, M1

*We call them the “ba chinja chinja”[throat-slitters].* —FGD1, M2

*We saw serious atrocities and these will stay in our memories for a long time*. —FGD2, F1

*The insecurity has now become permanently established there. They killed people there, including members of my family.* —FGD1, M2

*Me, I don't like to be reminded of this. We suffer a lot.* —FGD3, M2

The journey IDPs had followed to reach the camp was often challenging and circuitous, passing through multiple temporary dwelling places before arriving in their current camp:

*We spent nights outside in the bush during the armed attacks by those people.* —FGD5, F7

*In reality, when these people come to kill, you are just driven by a reflex to survive initially. And the next day, you ask yourself: now what? What do I do?* —FGD1, M1

*First, it's panic, you have to flee and you don't know who is where. You leave the house empty-handed, maybe with a child, and everybody has to flee. The next day, it's counting the dead and the damages. Then rapidly finding where to stay for security.* —FGD1, M3

*We passed through several areas, depending on the security situation. There was a lot of back and forth just attempting to restart a stable life.* —FGD1, M4

Loss of housing, assets, and livelihood meant that IDPs current condition was precarious:

*The war… a very bad thing. They attacked my village several times and we had to abandon everything, eventually arriving here.* —FGD3, M5

*Those fields are our guarantee for life.* —FGD1, M1

*…Our saving for the present and the future. It's our wealth, what keeps us alive, feeds us, pays for health care and school for our children.* —FGD1, M4

Some FGD participants expressed paralysis, hopelessness, and a sense of abandonment:

*On 1 side, the insecurity, and on the other, this corona—yes, we are scared. I'm just in shock. I can't say anything at the moment. And tell that government, there, that we are abandoned here.* —FGD5, F5

#### Population Movements In and Out of the Camp/Risk of Introducing COVID-19

Some FGD participants pointed out the insecurity and isolation of the camp that restricted travel:

*The area is very dangerous, they try to limit the movements.* —FGD4, Y3

*It's rare to visit others. We spend most of the time here or in the fields.* —FGD4, Y3

Others identified sources of visitors from outside the camp and noted that many IDPs move out of the camp for work on a daily basis.

*They [visitors] come from other camps or people who have fields that employ us to work in their fields.* —FGD2, F2

*No, in terms of leaving the camp, you can't count the number of times. If you stay here, the children will die of hunger. Many times a day to look for something to eat. To the market, to the fields, anywhere that you can find something.* —FGD6, F10

*There is a constant coming and going of people from outside the camp and vice-versa.* —FGD 6, F11

#### High Level of COVID-19 Awareness and Fear

There was a high level of awareness and fear of COVID-19, which was known as “corona”:

*A new disease and very severe. We are afraid of it and we pray that it stays away from us.* —FGD3, M7

*Concerning this corona, we have learned about this from afar. We have never seen a person sick with corona. But we have received teaching on corona*. —FGD5, F8

*We have learned that it kills mostly politicians and white people. We hope that this disease stays over there, away from us.* —FGD3, M6

As another severe viral epidemic, COVID-19 invited comparisons to the Ebola virus disease epidemic that had ravaged the region:

*We are very afraid because we have seen the families that lost their family members who died of Ebola.* —FGD4, Y1

*Ebola killed people, yes, but the radio talks of frightening numbers of deaths due to corona. Really very many.* —FGD6, F9

*Even with Ebola here, we went to church, to the market, but with corona, no. The churches are closed, and that's where we go for consolation, imploring God to protect us. But corona closed the churches. It's serious*. —FGD6, F10

#### Challenges Associated With Hand Hygiene in the Camp

Most FGD participants were aware of the recommendation for frequent handwashing as a prevention measure against COVID-19. However, soap and water were not readily accessible in the camp:

*You have to wash your hands. That's what they say, but we don't have water here.* —FGD4, Y2

*Our only source of water is the rain. We collect water when it rains and we keep it. We drink this water. When there is none left, our sisters go to the well to get water.* —FGD4, Y2

*There is a little stream about 100 m away. That's what we use for all our needs.* —FGD6, F9

*To wash our hands, we have water buckets but no soap and it's not enough because there are only 5 buckets for the whole camp [of approximately 800 people]*. —FGD6, F11

*They talk about masks, but if we don't even have soap, how can we ask for more? —FGD*2, F2

#### Impossibility of Physical Distancing in the IDP Camp

Housing was not conducive to physical distancing for many IDPs. Although many IDPs had individual family dwellings, some were housed in local school buildings, sleeping in classrooms.

*The director and state authorities allowed us to stay here. More and more people came to stay because there was space.* —FGD5, F5

*We don't pay anything for rent. It's free. —*FGD1, M1

*In the morning, we move our belongings outside until the end of classes. And at night, we bring back our things into the classrooms we occupy. But since the beginning of corona, we've stopped moving things in and out. We keep everything in the rooms where we sleep.* —FGD1, M3

Despite being accommodated by the school, tragically, IDP children did not attend classes:

*We stay with them outside, or else, they come with us to the fields nearby.* —FGD3, M6

*Where are we going to find money to pay the school fees? It's impossible. We are “wakimbizi” [refugees; those who fled], as they call us.* —FGD3, M5

A repeated theme was the inability to practice physical distancing because of crowded conditions, particularly sleeping quarters in which multiple families occupied a single classroom:

*Here, it's not possible “ku achana metre moya moya” [to stay 1 meter apart; to practice physical distancing]. If it comes here, we will all die. You have seen the conditions we live in. Our room measures 6 m by 5 m, and there are 5 families inside.* —FGD1, M4

*There is no soap, water is a problem, we sleep side by side. Everything is stacked against if this corona arrives here, even if we have, until now, escaped from the “ba chinja chinja” [throat-slitters].* —FGD1, M1

*We are crowded in classrooms like sardines. Isn't that awful?* —FGD6, F10

*One nongovernmental organization came here to educate us about corona. We asked the teacher to give us a practical demonstration. He just smiled! It's good to teach us, but going back, you should tell the people who sent you that it's not possible to avoid corona over there.* —FGD1, M1

*Do you see how we sleep? During the day, maybe, we can avoid touching each other, but at night we're in a small room. We're squeezed one against the other. It's not possible here.* —FGD2, F4

A repeated theme was the inability to practice physical distancing because of crowded conditions, particularly sleeping quarters.

#### Restoring Peace and Security Takes Priority Over Vaccine

Several respondents were willing to accept vaccination to prevent COVID-19 if a vaccine becomes available:

*I would receive it. For Ebola, people accepted the vaccine.* —FGD5, F6

Others bristled at the idea of a vaccine when more basic needs remain unmet:

*Our concern is safety. Even that vaccine doesn't matter to us. Let them keep it over there. Even if they vaccinate us, and we continue to live in these conditions, what's the point?* —FGD5, F5

*If security returns, we will protect ourselves against corona, we will respect all the measures, and it's only at that time that you can start talking about a vaccine or physical distancing. But in these conditions, I wouldn't accept this vaccine.* —FGD5, F5

In several FGDs, participants emphasized that COVID-19 prevention recommendations could best be implemented in a more stable, less crowded environment, such as their own homes. Reestablishing security in the region would allow IDPs to return where prevention could be practiced. Other prevention strategies were seen as context inappropriate or even futile:

*The government should bring back peace, we will go back to our homes and we will put into practice all that you have taught us. But it's impossible to prevent corona here.* —FGD1, M3

*These are measures that don't apply to us. The only medicine for us here or the only solution that can help us to fight corona here, is security. Bring back peace, and we'll go back home, where we live in good conditions, and we can respect these recommendations of 1m.* —FGD5, F7

*Me, I'll only be able to protect myself and my children when I'm at home. We have our own houses with plenty of space, like 6 rooms, but here it's 1 room. One room with several families. Each has his own activities during the day and you don't know who will bring you the disease.* —FGD6, F11

Reestablishing security in the region would allow IDPs to return where COVID-19 prevention recommendations could be practiced.

### Survey Questionnaire

Surveys were conducted between 25 and 29 May 2020. One IDP approached declined to participate in the questionnaire interview (165/166 [99%] participation rate). Two participants (1 IDP, 1 comparison) had never heard of coronavirus (307/309 [99%] awareness) and were excluded from the subsequent analysis. The final sample consisted of 164 IDPs (66 from Mwangaza; 44 Masosi, and 54 Luvangira) and 143 in the comparison group. There were 74 women (45%) among the IDPs surveyed and 57 women (40%) in the comparison group.

Thirty-five (21%), 82 (50%), and 47 (29%) of IDPs had lived in the camps for less than 1, 1–2, and more than 2 years, respectively. Sixty-six (41%) of families were temporarily sheltered in school buildings. Others lived in structures made from wood, thatch, and mud or brick walls with an iron sheet roof (Table 2). Demographic features, household (family) size, and asset ownership differed significantly between IDPs and the comparison group (Table 2). IDPs surveyed were older, had lower educational attainment, were more commonly farmers, were more commonly married, had a higher median household size, had lower household ownership of indicator assets (radio, cell phone, and bicycle), and had different housing structures than the comparison group (Table 2).

**TABLE 2.** Demographics of Survey Questionnaire Respondents Selected From 3 Internally Displaced Persons Camps in North Kivu, Democratic Republic of the Congo

	Overall(N=307)	IDPs (N=164)	Comparison (N=143)	*P* Value
**Demographics**				
**Age** [yr], median (IQR)	37 (24–55)	43 (28–58)	29 (22–45)	<.0001
**Sex, No. (%)**				.42
Male	176 (57.3)	90 (54.9)	86 (60.1)	
Female	131 (42.6)	74 (45.1)	57 (39.9)	
**Education, No. (%)**				<.0001
None	111 (36.2)	77 (47.0)	34 (23.8)	
Primary	113 (36.8)	67 (40.9)	46 (32.2)	
Secondary or above	83 (27.0)	20 (12.2)	63 (44.1)	
**Employment,** ^**a**^ **No. (%)**				<.0001
Farming	166 (54.1)	115 (70.1)	51 (35.7)	
Commerce/trade	20 (6.5)	1 (0.6)	19 (13.3)	
Health care worker	14 (4.6)	4 (2.4)	10 (7.0)	
Unemployed	77 (25.1)	37 (22.6)	40 (28.0)	
Other^a^	30 (9.8)	7 (4.3)	23 (16.1)	
**Marital status, No. (%)**				<.0001
Single	66 (21.4)	6 (3.7)	60 (42.0)	
Married	182 (59.3)	113 (68.9)	69 (48.3)	
Married (separated)	33 (10.7)	25 (15.2)	8 (5.6)	
Widowed	26 (8.5)	20 (12.2)	6 (4.2)	
**Household characteristics**				
Household size, median (IQR)	8 (6–10)	9 (7–11)	8 (6–10)	.007
Households with member aged >60 years, No. (%)	132 (43.0)	75 (45.7)	57 (40.0)	.33
**Household assets, No. (%)**				
Radio	158 (51.5)	52 (31.7)	106 (74.1)	<.0001
Cell phone	122 (39.7)	31 (18.9)	91 (63.6)	<.0001
Bicycle	50 (16.3)	10 (6.1)	40 (28.0)	<.0001
**Housing, No. (%)**				
Wood, thatch, mud materials	209 (68.8)	79 (49.1)	130 (90.9)	<.0001
Brick or wood walls and iron sheet roof	27 (8.9)	16 (9.9)	13 (9.1)	
School building	68 (22.4)	66 (41.0)	0	

Abbreviations: IDP, internally displaced person; IQR, interquartile range.

a Other employment included trades (mechanic, carpenter, shoemaker, tailor, mason, gardener), teacher, police officer, pastor, and taxi driver.

With respect to knowledge of COVID-19, fewer IDPs correctly identified signs and symptoms, and fewer recognized the potential for asymptomatic transmission (Table 3). Overall, 15% of IDPs had sufficient knowledge, versus 30% of the comparison group (OR=0.30; 95% confidence interval [CI]=0.17, 0.53; *P*<.0001). Other factors associated with low COVID-19 knowledge in bivariate analyses (*P*<.05) included younger age, larger household size, and lack of radio ownership. In a multivariable logistic regression model adjusting for these possible confounders, IDP status remained statistically significantly associated with lower knowledge (adjusted OR=0.17; 95% CI=0.082, 0.34; *P*<.0001).

**TABLE 3.** Survey Questionnaire Respondents’ Knowledge on COVID-19 Among Internally Displaced Persons, North Kivu, Democratic Republic of the Congo

	Overall (N=307) No. (%)	IDPs (N=164) No. (%)	Comparison (N=143) No. (%)	*P* Value
**Source of information on COVID-19**				
Local radio	278 (90.6)	140 (85.4)	138 (96.5)	.002
International radio	11 (3.6)	2 (1.2)	9 (6.3)	.03
Television	12 (3.9)	1 (0.6)	11 (7.7)	.002
Social media	28 (9.1)	2 (1.2)	26 (18.2)	<.0001
Church	40 (13.0)	28 (17.1)	12 (8.4)	.04
Friends	81 (26.4)	50 (30.5)	31 (21.7)	.11
No response	4 (1.3)	4 (2.4)	0 (0)	.13
**Recognition of illness**				
What are the signs and symptoms of COVID-19?^a^				
I don’t know	62 (20.2)	52 (31.7)	10 (7.0)	<.0001
Fever^b^	70 (22.8)	43 (26.2)	27 (18.9)	.16
Cough^b^	171 (55.7)	69 (42.1)	102 (71.3)	<.0001
Difficulty breathing^b^	109 (35.5)	46 (28.0)	63 (44.1)	.005
Sneezing	78 (25.4)	41 (25.0)	37 (25.9)	.96
Nasal congestion	140 (45.6)	59 (36.0)	81 (56.7)	.0004
Headache	67 (21.8)	46 (28.0)	21 (14.7)	.007
Fatigue	92 (29.9)	38 (23.2)	54 (37.8)	.008
Joint pain	80 (26.1)	29 (17.7)	51 (35.7)	.0006
Muscle pain	27 (8.8)	16 (9.8)	11 (7.7)	.66
Loss of appetite	14 (4.6)	6 (3.7)	8 (5.6)	.59
Diarrhea	25 (8.1)	18 (11.0)	7 (4.9)	.08
Constipation^c^	2 (0.7)	2 (1.2)	0 (0)	.50
Bleeding^b^ ^,^ ^c^	25 (8.1)	14 (8.5)	11 (7.7)	.95
**Asymptomatic spread**				
COVID-19 can be transmitted by someone with no symptoms.^b^	146 (47.6)	60 (36.6)	86 (60.1)	<.0001
**Misconceptions**				
COVID-19 can be transmitted by mosquitos.^b^ ^,^ ^c^	125 (40.7)	67 (40.9)	58 (40.6)	.19
COIVID-19 can be prevented by eating spicy food.^c^	21 (6.8)	11 (6.7)	10 (7.0)	.90
**Sufficient knowledge of COVID-19**				
Knew key symptoms, did not endorse misconceptions	79 (25.7)	24 (14.6)	55 (38.5)	<.0001

Abbreviations: COVID-19, coronavirus disease; IDP, internally displaced person.

a If participant answered “I don't know,” no further symptoms were solicited. Otherwise, multiple answers were allowed.

b Used to assess sufficient knowledge of COVID-19.

c Number (percentage) of participants who erroneously endorsed these incorrect signs, symptoms, or statements.

Attitudes and practices toward COVID-19 prevention are shown in Tables 4 and 5, respectively. Despite widespread agreement (89%) that physical distancing was important to prevent COVID-19, a higher proportion of IDPs than individuals in the comparison group reported close contact with someone outside the family in the past 24 hours and a higher proportion had shaken hands with at least 1 person (Table 5).

**TABLE 4.** Survey Questionnaire Respondents’ Attitudes^a^ Toward COVID-19 Among Internally Displaced Persons, North Kivu, Democratic Republic of the Congo

	Overall (N=307) No. (%)	IDPs (N=164) No. (%)	Comparison (N=143) No. (%)	*P* Value
**Affective response**				
COVID-19 is a serious illness.	301 (98.0)	163 (99.4)	138 (96.5)	.03
I am afraid of COVID-19.	300 (97.8)	161 (98.2)	139 (97.2)	.08
**Reaction to control measures**				
Physical distancing is important to prevent COVID-19.	278 (90.6)	146 (89.0)	132 (92.3)	.42
People should be willing to give up their daily duties to stop the spread of COVID-19.	243 (79.2)	120 (73.2)	123 (86.0)	.14
**Disinformation**				
It is hard to distinguish which information I hear about COVID-19 is true, false, or just a rumour.	244 (79.5)	126 (76.8)	118 (82.5)	.61
**Institutional trust**				
I trust the government.	207 (67.4)	117 (71.3)	90 (62.9)	.09
There is a lot of corruption in the government.	123 (40.1)	70 (42.7)	53 (37.1)	.53
**Rumors**				
COVID-19 was created in a Chinese laboratory.	58 (18.9)	31 (18.9)	27 (18.9)	.88
COVID-19 is a conspiracy created to vaccinate everybody.	37 (12.1)	13 (7.9)	24 (16.8)	.03

Abbreviations: COVID-19, coronavirus disease; IDP, internally displaced person.

a Participants were asked to rank agreement with the statements on a 5-point Likert scale, with possible answers “strongly agree,” “agree,” “neutral,” “disagree,” “strongly disagree,” or “I don't know.” Numbers are n (%) of participants who agreed or strongly agreed with the statements.

**TABLE 5.** Survey Questionnaire Respondents’ Practices With Respect to COVID-19 Prevention Among Internally Displaced Persons, North Kivu, Democratic Republic of the Congo

	Overall (N=307) No. (%)	IDPs (N=164) No. (%)	Comparison (N=143) No. (%)	*P* Value
**Prevention practices**				
In the past 2 weeks, have you done anything to protect yourself from COVID-19?				
No	137 (44.6)	77 (47.0)	60 (42.0)	
Yes	168 (54.7)	85 (51.8)	83 (58.0)	.39
If so, what?^a^				
Wash hands	149 (88.6)	78 (91.7)	71 (85.5)	.30
Stay >2 m from others	75 (44.6)	35 (41.2)	40 (48.2)	.45
Avoid touching face	38 (22.6)	24 (28.2)	14 (16.8)	.11
Stay home	31 (18.5)	10 (11.8)	21 (25.3)	.04
Use disinfectant	10 (6.0)	6 (7.1)	4 (4.8)	.75
Wear mask	8 (4.8)	3 (3.5)	5 (6.0)	.49
Take medicines without prescription	2 (1.2)	2 (2.4)	0 (0)	.50
Change diet	1 (0.6)	1 (1.2)	0 (0)	>.99
**Physical distancing**				
Apart from family, have you come in close (<2 m) contact with anyone in the past 24 hours?				
Yes	195 (63.5)	115 (70.1)	80 (55.9)	.01
How many people did you shake hands with in the past 24 hours (not counting family members)?				
0	155 (50.5)	77 (47.0)	78 (54.5)	
1 to 5	71 (23.1)	31 (18.9)	40 (28.0)	.02
>5	81 (26.4)	56 (34.1)	25 (17.5)	
**Barriers to prevention**				
What has prevented you from fully protecting yourself from COVID-19?				
Lack of soap	243 (79.2)	150 (91.5)	93 (65.0)	<.0001
Lack of water	193 (62.9)	110 (67.1)	83 (58.0)	.11
Insufficient income	67 (21.8)	32 (19.5)	35 (24.5)	.38
Lack of masks	55 (17.9)	25 (15.2)	30 (21.0)	.26
Lack of information	51 (16.6)	24 (14.6)	27 (18.9)	.41
Lack of disinfectant	46 (15.0)	21 (12.8)	25 (17.5)	.34
Lack of availability of these items	31 (10.1)	18 (11.0)	13 (9.1)	.71
High prices of these items in the market	46 (15.0)	15 (9.2)	31 (21.7)	.004
Lack gloves	18 (5.9)	11 (6.7)	7 (4.9)	.66
I can fully protect myself against COVID-19	28 (9.2)	7 (4.3)	21 (14.7)	.003

Abbreviations: COVID-19, coronavirus disease; IDP, internally displaced person.

a Among respondents who had done something to protect against COVID-19.

IDP respondents indicated that movements in and out of the camp were frequent. By self-report, 83 (61%), 62 (38%), and 19 (12%) left the camp on a daily, weekly, and monthly basis, respectively. In addition, 107 (65%) of IDPs had received a visitor from outside the camp in the past month. Since the pandemic began, IDPs reported leaving the camp less frequently than before in 84 (52%), more than before in 33 (20%) and about the same as before in 46 (28%) of cases.

## DISCUSSION

Our study is unique among COVID-19 KAP surveys to date for its focus on a displaced population with extreme resource limitations. Other KAP surveys included health care workers^28^ ^,^ ^45^ ^–^ ^47^ or residents of high-income countries with markedly different demographics than our study (e.g., 62% of U.S.^48^ and 64% of Chinese participants^49^ had a bachelor’s degree or higher, compared to 47% of IDPs in our study who had no formal education at all). Given the radically different challenges of COVID-19 prevention in IDP camps, this study fills a gap in available data from a neglected and isolated population. IDPs differed from neighboring Congolese residents in terms of larger household size (including 46% of families with a member over the age of 60), more extreme poverty, lower educational attainment, less access to information through media and internet, less COVID-19 specific knowledge, lower rate of physical distancing, and reduced access to hand hygiene. These factors, as well as the high mobility of IDPs, leaving and reentering the camp daily for subsistence labor, establish their vulnerability to COVID-19.

Given the radically different challenges of COVID-19 prevention in IDP camps, this study fills a gap in available data from a neglected and isolated population.

### COVID-19 Knowledge

IDPs and the comparison group both identified local radio as their major source of information on COVID-19 (Table 2). Radio, television, and social media were more common sources of information among the comparison group, whereas church was a more common source among IDPs (Table 2). Other studies in LMICs (Pakistan,^45^ Uganda,^28^ and Vietnam^50^) showed that health care workers accessed World Health Organization or ministry of health websites (83%–88%), social media (74%–91%), radio or television (46%–79%) for their COVID-19 information, preferences which reflect major differences in education level, employment activities, and access to internet from the IDPs in our study.

Knowledge of COVID-19 was poor in IDPs versus the comparison group (Table 3). Using a similar questionnaire item, 98% and 93% of health care workers in Uganda identified fever and cough as symptoms of COVID-19,^28^ compared to 26% and 42% of IDPs, respectively, in our study. Gastrointestinal symptoms were less frequently identified by both Ugandan health care workers (35%)^28^ and IDPs (11%). Misconceptions around COVID-19 transmission (incorrectly endorsing mosquito-borne transmission) were common in both IDPs (54%) and the comparison group (64%) in our study.

Fear of COVID-19 was expressed by 98% of survey respondents, similar to previous observations of high anxiety scores in another survey from Iran.^47^ Surprisingly, many FGD participants considered that COVID-19 was even more severe than Ebola virus disease (in fact, the case fatality rate of Ebola virus disease is more than 60%,^51^ compared to less than 2% for COVID-19^52^). Public health messages about the severity of COVID-19 appear to be widely accepted and believed, with FGD participants citing the high number of deaths in wealthy “white” countries and the closing of churches as evidence of danger. Although mistrust in the government (39%), belief in corruption (42%), belief in conspiracy theories (44% and 22%) were prevalent, endorsement of these views did not appear to be associated with prevention practices. This contrasts with surveys of attitudes toward Ebola virus disease in the same area, in which mistrust, rumors, and misinformation were associated with passive and active resistance to control measures.^17^ ^,^ ^41^

Many FGD participants considered that COVID-19 was even more severe than Ebola virus disease.

### COVID-19 Prevention Efforts

COVID-19 prevention practices vary widely between geographic areas and demographic groups. For example, 98% of Chinese residents at the beginning of the pandemic wore masks when going out^49^ compared to 24% of U.S. residents.^48^ Mask use was reported by 3.5% of IDPs and 6% of the comparison group, highlighting the lack of personal protective equipment in this setting. Other measures more readily available to IDPs were handwashing (practiced by 98%), distancing from others (48%), and avoiding touching the face (28%), which were reported in proportions similar to the comparison group.

Movement of populations contributes to the spread of COVID-19. In a large refugee camp in Bangladesh, aid workers who enter and leave the camp daily are expected to be the most likely sources of introduction of COVID-19 into the camp.^12^ In the IDP camps in our study, the conspicuous lack of aid workers reflects the isolated and hazardous environment, as well as the neglected status of the IDPs. However, 61% of IDPs left the camp on a daily basis, and 65% had received a visitor in the past month. Staying home was practiced less often among IDPs than among the comparison group (*P*=.039, Table 5). These frequent movements represent opportunities to introduce COVID-19 into the camp. FGD participants explained that daily labor in neighboring fields or trips to the market were imperative to provide for family needs. Thus, unless food security can be assured by other means, restriction of movements to prevent COVID-19 is not viable in the IDP camps studied.

Among IDPs who had taken action to prevent COVID-19, hand hygiene was practiced by 92%. However, the most commonly listed barrier to prevention was lack of soap (92% of IDPs, versus 65% of the comparison group), followed by lack of water (67% of IDPs). Distribution of soap to households in a refugee camp increased handwashing by more than 30% and reduced diarrheal illness in a previous study.^53^ In Nigeria, COVID-19 control efforts included sensitization campaigns on handwashing were followed by the distribution of soap to IDPs in Borno State.^13^ Inspired by these examples, and responding to the near-universal lack of soap identified in our survey, we included soap distribution in our community feedback efforts.

Avoiding physical contact with others is emphasized as a COVID-19 prevention measure. The majority (89%) of IDPs agreed or strongly agreed that this was an important control measure (Table 4), but 70% had come in close contact with someone other than a family member (versus 56% of the comparison group, *P*=.014, Table 5). The impossibility of physical distancing in the camp, noted by previous authors,^10^ ^–^ ^12^ was repeatedly emphasized in FGDs. Sleeping quarters were highly congested, with several families often sleeping in a single classroom. In high-income countries, where shelter-at-home recommendations are more feasible, adherence to physical distancing recommendations remains variable. In the United States, 30% of people reported attending gatherings with more than 50 people (contrary to public health advice),^48^ compared to only 3.6% of Chinese survey respondents.^49^ In our study, 19% of IDPs had shaken hands with 1–5 people in the past 24 hours, and 34% with more than 5 people, which was statistically higher than the comparison group (*P*=.023, Table 5). In contrast, 83% of Ugandan health care workers avoided shaking hands due to COVID-19.^28^ Given challenges with hand hygiene and physical distancing in the camps, we speculated that IDPs may have felt disempowered to make even small efforts to reduce physical contact with others.

Given challenges with hand hygiene and physical distancing in the camps, we speculated that IDPs may have felt disempowered to take small efforts to reduce physical contact with others.

Acceptance of a hypothetical COVID-19 vaccine was high (92%) in a study of Vietnamese health workers.^50^ In our FGDs, some participants were willing to accept vaccination as a control strategy, whereas others pointed to futility and inappropriateness of what appeared to them as a stopgap solution, when the overwhelming problem was displacement from their homes.

Expressions of futility or fatalism as expressed by FGD participants in our study are noteworthy and may reflect learned helplessness or loss of self-efficacy among IDPs under extraordinarily difficult living conditions. The theory of learned helplessness^54^ ^,^ ^55^ describes pessimistic beliefs about the efficacy of one's actions and the likelihood of obtaining future rewards. The theory has explanatory power among refugees in other contexts, such as risky sexual behavior among victims of sexual or gender-based violence.^56^ Similarly, the concept of self-efficacy^57^ refers to the degree of externality in control attribution.^58^ Low self-efficacy is associated with a fatalistic orientation, as exemplified by a FGD participant’s response. These theoretical frameworks may explain, at least in part, initially puzzling findings such as rejection of a hypothetical vaccine among some FGD respondents and high levels of hand shaking despite awareness and fear of COVID-19.

### Limitations

Our study has several limitations. Our survey tool was not validated against a gold standard instrument for the measurement of COVID-19-related KAP among IDPs. However, we took several steps to optimize the validity of the survey: (1) contextually relevant questionnaire items using past surveys from other LMICs and from North Kivu; (2) tacit understanding of the local language and culture by our study team; and (3) implementation of the questionnaire as a verbal interview by local Congolese health workers to allow explanation of questions. The sampling strategy for IDPs and the comparison group was not a fully random sample due to lack of detailed census information. Instead, for IDPs we used geospatial sampling^34^ from 3 displacement camps. For the comparison group, we used maximum variation sampling, based on demographic features (age, sex, occupation, and educational attainment). These nonprobability sampling methods are widely used,^37^ but findings may not be representative of the entire IDP population. Therefore statistical inferences should be interpreted with caution and should be confirmed in studies with a fully random sample of the population of interest (IDPs in North Kivu, DRC). For our primary analysis (COVID-19 knowledge among IDPs versus the comparison group), we adjusted for differences in demographic variables between groups in a multivariable analysis to mitigate the effect of confounding. Similarly, FGDs participants represented a small number of IDPs in the camp; however, saturation of themes was quickly achieved, suggesting the breadth and diversity of viewpoints in the camps was captured.

## CONCLUSION

In summary, our findings provide a snapshot of IDP camps as they brace for COVID-19. Awareness and fear of COVID-19 was high among IDPs, but only 15% had comprehensive knowledge of the disease. Significant barriers to implementing COVID-19 prevention measures exist in IDP camps, including crowded sleeping quarters, frequent close contact with non-family members, movement in and out of the camp for work, and lack of access to hand hygiene. Poignantly, IDPs spoke of a desire for peace and a return to their homes, where they could capably prevent COVID-19 themselves. These data from a hard-to-reach population in a zone of insecurity provide a rare glimpse of the desperate conditions under which IDPs survive, leaving them vulnerable to COVID-19. These results call for an ethical, inclusive approach to the global pandemic that leaves no one behind, just as COVID-19 will not respect borders and will not leave behind refugees and IDPs.^6^

## RECOMMENDATIONS

These specific recommendations follow from our findings:

- IDPs should be provided with adequate facilities and consumables to implement recommended COVID-19 precautions. These include ample water and soap for hand hygiene and face masks.
- Additional space and housing should be made available to allow IDPs to practice physical distancing, particularly within sleeping quarters. Separate dwellings (e.g., tarpaulin tents) for individual families should be provided. Multiple families sleeping in a classroom (as currently observed) is discouraged.
- Although challenging, restoration of peace by controlling armed conflict in the area is a chief priority for IDPs and would allow a safe return to their ancestral homes where they could more adequately practice COVID-19 prevention.

## Notes

**En français**

La prévention de COVID-19 dans un camp des déplacés internes dans une zone d’insécurité au Nord Kivu, République Démocratique du Congo: une étude avec méthodes mixtes

**Message clé**

Les déplacés internes (DI) à cause d’un conflit armé constituent une population négligée, vulnérable à la pandémie de COVID-19. Cette étude avec méthodes mixtes donne une rare perspective sur les défis auxquels font face les DI à l’Est de la République Démocratique du Congo devant la menace de COVID-19.

**Résumé** **Introduction:** La pandémie de COVID-19 présente une sérieuse menace aux réfugiés et aux déplacés internes (DI). Nous avons étudié les connaissances, attitudes, et pratiques vis-à-vis COVID-19 parmi les DI dans une zone d’insécurité à l’Est de la République Démocratique du Congo (RDC).

**Méthodes:** Étude avec méthodes mixtes pour la collecte et analyse de données qualitatives (discussions en groupe, DG) et quantitatives (sondage avec questionnaire de 52 éléments).

**Résultats:** Des DG (23 participants au total) et un sondage (164 DI de trois camps de déplacés et 143 témoins d’un village voisin) ont été organisés en mai, 2020. Les DI étaient statistiquement plus susceptibles d'avoir une plus grande taille de ménage, une pauvreté extrême, un niveau d'éducation inférieur et un accès plus faible à l'information via les médias et l’internet (P <0,05 pour toutes les comparaisons). Les PDI avaient un niveau élevé de sensibilisation (99%) et de peur (98%) du COVID-19, mais des connaissances spécifiques plus faibles (15% de connaissances suffisantes contre 30% parmi les témoins, P <0,0001), une différence qui est restée significative dans un modèle multivariable ajusté pour les effets confondants. Les DI avaient plusieurs défis quant à la mise en œuvre des recommandations pour prévenir le COVID-19. La distanciation physique était impossible dans leurs abris coincés et 70% des DI ont répondu qu’ils ont été en contact étroit avec une personne autre qu'un membre de la famille au cours des dernières 24 heures (contre 56% des témoins, P=0,014). Les DI devaient souvent sortir du camp pour subvenir à leurs besoins alimentaires, ce qui pourrait permettre l’introduction de COVID-19 dans le camp. 61% des DI sortaient du camp quotidiennement, et 65% avaient eu un visiteur dans le mois précédent. Malgré l'acceptation de l'hygiène des mains pour la prévention, 92% manquaient de savon (contre 65% des témoins, P <0,0001). Les DI cherchaient la paix et un retour au village natal encore plus que d’autres mesures de prévention telles qu’un vaccin contre COVID-19.

**Conclusions:** Ces résultats fournissent des preuves empiriques soutenant la vulnérabilité des DI au COVID-19 et appellent à l'action pour protéger les populations déplacées négligées.

## Notes

**Peer Reviewed**

**First published online:** December 4, 2020

**Funding:** The Association for Health Innovation in Africa (AFHIA) provided funding for this study.
